# Case Report: Morphological Characterization and Long-Term Observation of Bilateral Sequential Internal Mammary Artery Aneurysms in a Patient With Confirmed FBN1 Mutation

**DOI:** 10.3389/fcvm.2021.697591

**Published:** 2021-06-16

**Authors:** Roland Stengl, Bence Ágg, Bálint Szilveszter, Kálmán Benke, Noémi Daradics, Bernadett Ruskó, Borbála Vattay, Béla Merkely, Miklós Pólos, Zoltán Szabolcs

**Affiliations:** ^1^Heart and Vascular Center, Semmelweis University, Budapest, Hungary; ^2^Hungarian Marfan Foundation, Budapest, Hungary; ^3^Department of Pharmacology and Pharmacotherapy, Semmelweis University, Budapest, Hungary; ^4^MTA-SE Cardiovascular Imaging Research Group, Heart and Vascular Center, Semmelweis University, Budapest, Hungary

**Keywords:** Marfan syndrome, aneurysm, internal mammary arteries, follow-up, tortuosity, case report

## Abstract

Marfan syndrome (MFS) is a genetically determined connective tissue disorder that leads to ocular, skeletal, and severe cardiovascular involvement. High mortality of MFS is associated with aortic dissection and aneurysm characteristic to the syndrome. In MFS, only a few cases of peripheral arterial involvement have been reported so far, mostly without a genetically confirmed diagnosis. We report a 41-year-old MFS patient with a saccular pearl-string-like aneurysm on the right internal mammary artery (RIMA) and a single aneurysm on the left internal mammary artery (LIMA). To our knowledge this is the first reported case on internal mammary artery aneurysms with this special morphology and with follow-up and blood pressure control as primary therapeutic approach in a patient with genetically confirmed MFS. The aneurysms with the above described morphology first appeared as small aneurysms on a CT scan 6 years after a cardiac operation. Due to the lack of guidelines, based on the asymptomatic state of the patient, the increased tortuosity of the affected vessels and the history of prior cardiac surgery, we decided to closely monitor these aneurysms with blood pressure control and without carrying out any interventions. On the CT scans done 3, 11, 12, 17, and 32 months after identifying the aneurysms, no progression of these structures was detected. Our findings confirm the possibility of the occurrence of internal mammary artery aneurysms in patients with *FBN1* mutation and we believe that monitoring these aneurysms with blood pressure management can be a suitable option in selected cases.

## Introduction

Marfan syndrome (MFS) is a relatively common genetically determined connective tissue disorder with a prevalence of about 2–3 per 10,000 individuals (1). A wide variety of mutations in the fibrillin-1 (*FBN1*) gene can be found in the majority of MFS patients (2, 3). The mutations in the *FBN1* gene, through the failure of fibrillin-1, a constituent of elastic fibers and microfibrils, cause decreased elastin content and fragmentation of elastic fibers. In addition to the obvious structural role of fibrillin-1, it was demonstrated that microfibrils also have a critical role in the regulation of transforming growth factor beta signaling that affects the expression of many proteins in the connective tissue. These molecular alterations result in an inelastic and tear-prone connective tissue, which can cause skeletal, ocular, skin, and cardiovascular abnormalities typical to the syndrome (4).

Aortic aneurysm and dissection are well-known life-threatening manifestations of MFS (1). Other large and medium sized arteries, as the iliac, carotid and ulnar arteries, can also be affected in MFS leading to aneurysms and rupture (5–7). However, there have been only a couple of reports about small sized arteries in MFS. Internal mammary artery (IMA) aneurysm in MFS have been observed only a few times, nevertheless it is of outstanding importance as in case of rupture it can lead to hemothorax and life-threatening hemorrhage (8–11). However, invasive procedures used to treat these aneurysms can also lead to complications (11), open surgical repair always carries risks and the use of endovascular techniques in MFS patients has been controversial (12). Due to their extremely rare occurrence, guidelines on their management are not available. We describe a patient with genetically confirmed MFS who meets the criteria of the revised Ghent nosology presenting with IMA aneurysms with a special morphology that are being monitored with blood pressure control and without any interventions.

## Case Description

A 41-year-old male patient with known MFS has been followed-up in our institution with extensive cardiovascular complications.

The patient has a systemic score of eight points according to the revised Ghent nosology. His skeletal features include pectus carinatum, wrist- and thumb sign, reduced extension of the elbows, hypermobile joints, and retrognathia. Ectopia lentis and myopia >3 diopters account for his ocular involvement. As discussed below, he also has severe cardiovascular manifestations.

His family history is positive for MFS. His father did not show similar clinical features, but he died from aortic dissection at the age of 47 years. The patient's mother and sister share similar anthropometric features with the patient.

Because of the clinical diagnosis of MFS, the patient took part in our ongoing genetic testing program for MFS and related disorders. A next-generation sequencing (NGS)-based multi-gene panel was applied to screen the following nine genes: *ACTA2, COL3A1, FBN1, KCNN1, MYH11, SMAD3, TGFB2, TGFBR1*, and *TGFBR2* (13). A pathogenic mutation in the *FBN1* gene was detected, confirming the diagnosis of MFS.

**Figure 1:**
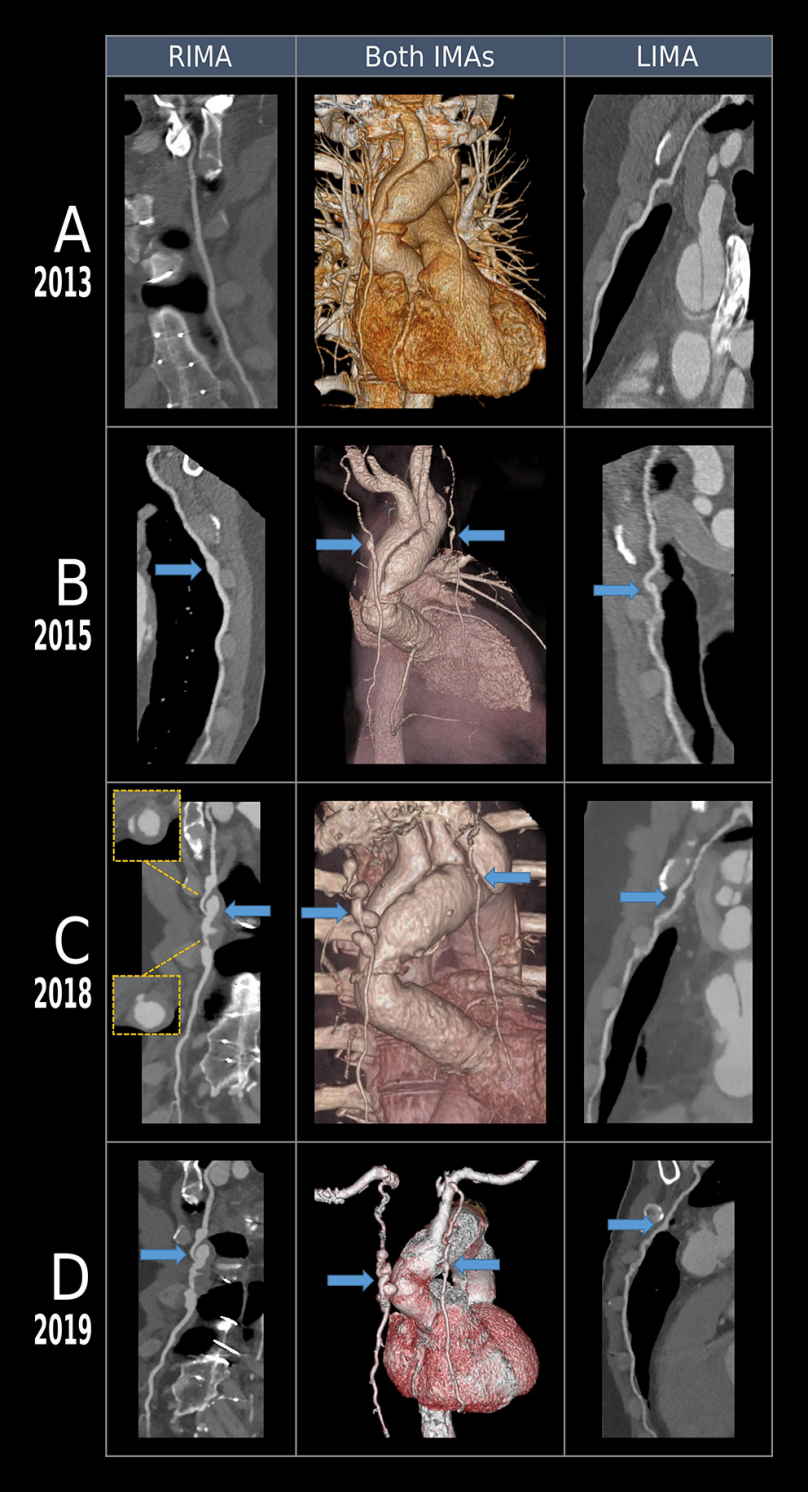
Volumetric and multiplanar reconstructions of the aorta, left and right internal mammary artery (IMA) of a Marfan syndrome patient after Bentall procedure. 256-slice CT angiography of the chest was performed at three different timepoints. **(A)** In 2013, left and right IMA-s were intact without any signs of aneurysms. **(B)** In 2015, both arteries demonstrated a small dilatation (~0.5 cm) in the mid-section. **(C)** Follow-up scans after 3 years revealed a saccular pearl-string-like aneurysm of the right IMA with thrombus on the walls, an aneurysm of the left IMA and increase in diameter of the aortic arch. **(D)** Follow-up CT done in 2019 shows no progression of the aneurysms.

The patient underwent two cardiac surgeries. Due to his gradually expanding aorta that led to aortic insufficiency, and known familial MFS history with a father who suffered from aortic dissection, the patient underwent Bentall procedure in November 2009. During the operation the surgeons faced chronic aortic dissection. Postoperative period was uneventful, the patient was started on antihypertensive drugs, namely metoprolol 2 × 25 mg and ramipril 2 × 5 mg, and he was also prescribed acenocoumarol 2 mg with the aim of anticoagulation. The patient had been followed-up regularly with echocardiography and as part of his evaluation, he had a contrast enhanced chest computed tomography (CT) scan in February 2013 (Figure 1A), which showed that the aortic arch expanded distally to the conduit. An intimal flap could also be observed in the aortic lumen, but there were no signs of IMA aneurysms.

On the subsequent CT in June 2015 (Figure 1B) the intimal flap and the degree of aortic dilation did not change significantly compared to the condition in 2013. However, new aneurysms appeared with a diameter of 0.5 cm in the mid-section of IMA on both sides, which were only identified retrospectively, they were not reported at that time. These aneurysms could not be observed on the previous CT scans, they probably started to develop 6 years after the procedure, therefore, they are more likely to be caused by the underlying connective tissue disorder than being the consequence of the previous cardiac operation.

In February 2018 a control CT (Figure 1C) revealed the progression of the aortic dilatation and four sequential saccular pearl-string-like aneurysms could be observed on the right IMA (RIMA) in the level of the body of the sternum with a growing diameter of 10–19 mm. Some of them had partial wall thrombosis. Symmetrically, on the left IMA (LIMA) a 12 mm expansion was observed. Despite their initial appearance on the previous CT scan, they were first discovered on the CT done in 2018, as they reached a notable size only by this time. Due to the lack of guidelines on the management of IMA aneurysms, given the asymptomatic state of the patient, the increased tortuosity of the affected vessels seen on the CT scans (Figure 1) and the expected difficult anatomic situation after the prior cardiac surgery, we decided to apply a close follow-up with blood pressure control to avoid the probable complications of an intervention. As IMA aneurysms are extremely rare, currently there are not enough experience in our department with their endovascular treatment, therefore it could result in serious complications, especially in patients with connective tissue disorder. Furthermore, the case report by Awais et al. (11) supported our decision as they applied follow-up after a failed intervention attempt in case of bilateral IMA aneurysms in a patient with Marfan syndrome. However, it does not exclude the possibility of carrying out an endovascular intervention in case of symptom onset or progression of the aneurysms. Blood pressure control involved antihypertensive drugs and lifestyle restrictions to avoid activities with relevant blood pressure rises.

The patient had been asymptomatic until March 2018, when he presented with a sudden chest pain and an extended dissection of the aorta to the origin of the right common iliac artery was revealed by a CT scan. Due to the high risk of the patient, a multidisciplinary team decided against acute surgery, therefore after stabilization and antihypertensive treatment, the patient was assigned for an elective aortic reconstruction in the following year. He was prescribed the following medications: amlodipine 2 × 10 mg, ramipril 2 × 10 mg, urapidil 3 × 90 mg, doxazosin 2 × 4 mg, bisoprolol 5 mg, hydrochlorothiazide 25 mg, nitroglycerin patch 10 mg, acenocoumarol, and pantoprazole 40 mg.

The CT scans done 3, 11, and 12 months after identifying the aneurysms did not show any progression of these structures. In February 2019, the patient underwent the planned elective aortic reconstruction procedure in the form of total aortic arch replacement with elephant trunk prosthesis. The aneurysms were not treated during the surgery due to severe adhesions at the surgical site caused by the previous operation, posing a higher risk for possible complications. Furthermore, the patient presented wound and bone healing problems after his first heart surgery, the recurrence of which we wanted to avoid. Therefore, we decided against a bilateral intervention, which could have worsened the blood supply to the area, leading to healing issues. It is also important to note that the patient had pectus carinatum, which further complicated the case. The surveillance CT scan done in July 2019, 17 months after identifying the saccular pearl-string-like RIMA aneurysms and the LIMA aneurysm, did not show any progression of these structures. No progression of the aneurysms was observed in the most recent CT scan done in October 2020, 32 months after their initial detection.

**Figure 2:**
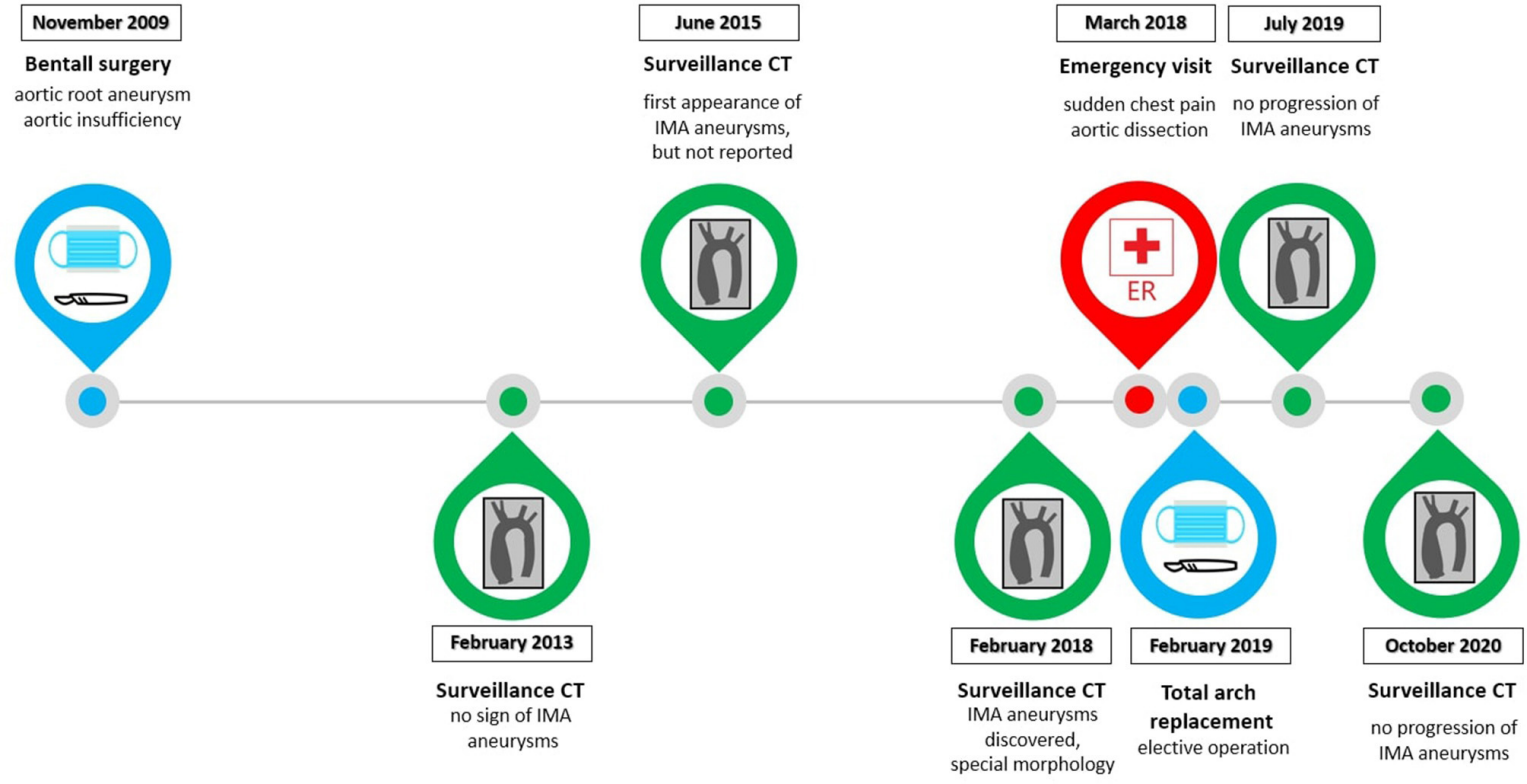
The timeline demonstrates the main episodes of patient care. The key steps of surveillance CT scans are shown, the last one was done in October 2020. The two surgeries and the emergency care were also important aspects in the patient's case.

Figure 2 shows the main episodes of patient care.

Hypercholesterolemia is the only known cardiovascular risk factor of the patient.

**Figure 3:**
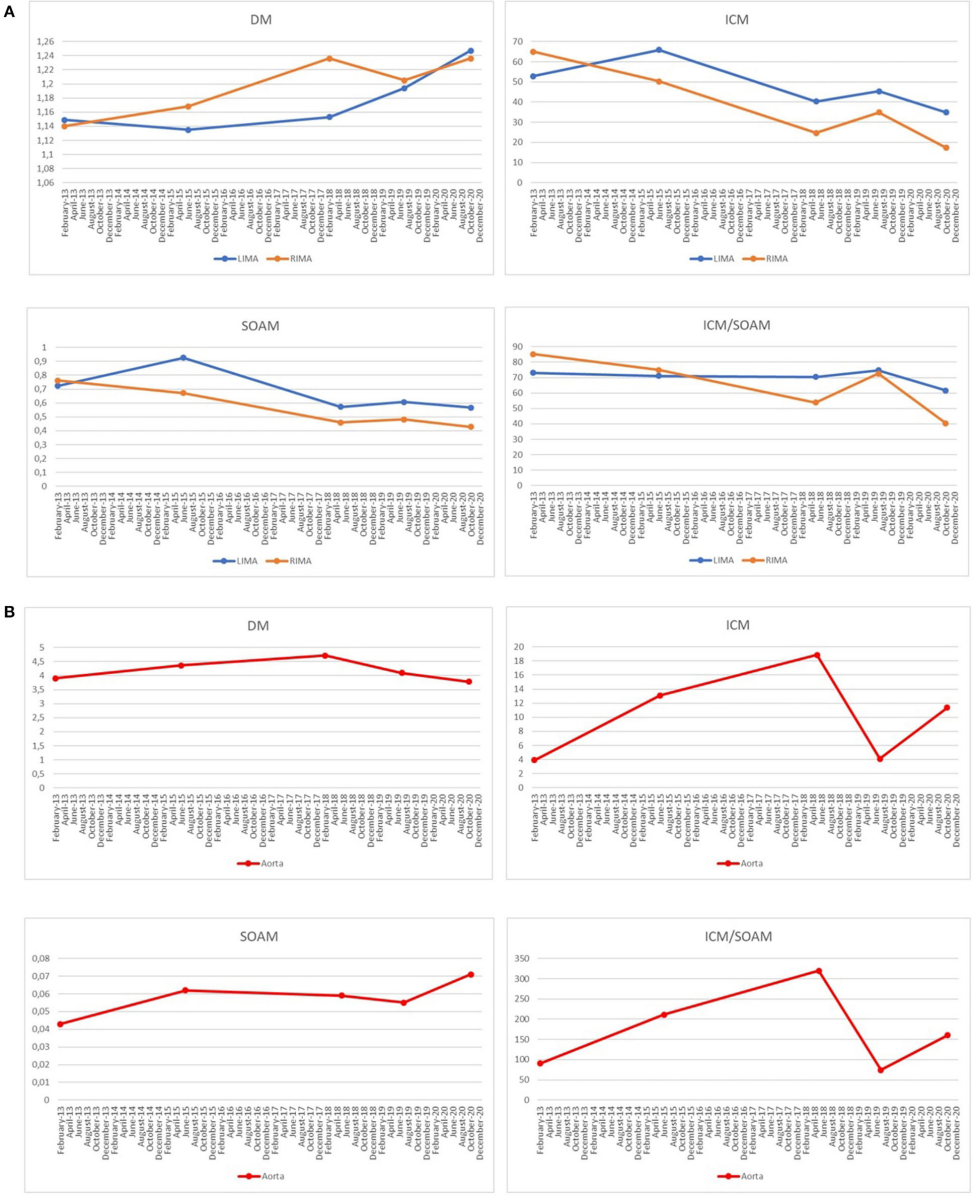
**(A)** A progression of the tortuosity of the right internal mammary artery (RIMA) and the left internal mammary artery (LIMA) demonstrated by an increase in the Distance Metric (DM) value can be observed. The other parameters used to assess the tortuosity of a vessel are Inflection count metric (ICM), which is sensitive to higher amplitude structures, Sum of angles metric (SOAM), which is increased in case of higher frequency structures and ICM/SOAM. In this case, they are less conclusive as they all show a decreasing tendency. **(B)** The thoracic aorta shows an increasing tortuosity with increasing amplitude and frequency of the curves demonstrated by the overall rise of the ICM, SOAM, and ICM/SOAM parameters.

We assessed the tortuosity of the internal mammary arteries and the thoracic aorta (SE RKEB 72/2018) by analyzing the distance metric (DM), inflection count metric (ICM), sum of angles metric (SOAM) and ICM/SOAM, as previously described (14). We performed ECG-gated 256-slice CT of the chest with iodinated contrast agent. Centerlines of the thoracic aorta, the left and right IMA were extracted using dedicated software tools (MEDIS Qangio CT 3.1) and exported in text format containing the 3-dimensional vessel coordinates for tortuosity assessment (MEDIS Qangio 3D Workbench 0.8). Parallel to the progression of the aneurysms, an increase in the DM can be observed in case of the RIMA and the LIMA, which demonstrates a progression of the tortuosity of these vessels (Figure 3A). At the same time the thoracic aorta demonstrates an overall rise in the ICM, SOAM and ICM/SOAM parameters, meaning an increasing tortuosity with increasing amplitude and frequency of the curves, dominated by the rise in amplitude as suggested by ICM/SOAM (Figure 3B).

Currently, the patient has been observed after cardiac operations and due to his extensive bilateral IMA aneurysms. At present the patient is asymptomatic. On physical exam no abnormalities in relation to the IMA aneurysms can be found. After the second operation, during the follow-up period, the antihypertensive medications of the patient were adjusted according to his blood pressure values and at the time of writing the case report the patient was taking the following blood pressure medications: amlodipine 2 × 5 mg, ramipril 2 × 5 mg, doxasozin 1 × 4 mg, urapidil 3 × 30 mg, and bisoprolol 1 × 5 mg.

We continue to closely monitor the patient with appropriate blood pressure control. In case of progression of the aneurysms or onset of aneurysm-related symptoms we are going to carry out an intervention, preferably by means of endovascular approach.

## Discussion

We described the case of a patient with a genetically confirmed diagnosis of MFS who was presented with bilateral IMA aneurysms, of which the RIMA aneurysms showed a saccular pearl-string-like morphology. A similar morphology has been reported only in a patient with a *SMAD3* mutation (10). In our case, MFS was confirmed with genetic testing. The assessment of the tortuosity of the affected vessels and the thoracic aorta, the morphological characterization of the aneurysms, their evolution, and a proposed conservative therapeutic approach were also presented. The development of the aneurysms can be followed on the CT scans (Figure 1), which show that they were not present in 2013, 4 years after the first cardiac surgery, they only appeared in 2015 and they reached this special morphology in ~3 years, and they have not progressed since then.

MFS is characterized by aortic aneurysm formation, mainly affecting the aortic root (1). On the other hand, as only a few case reports and some small-scale studies on patients with genetically established diagnosis of MFS have been published in the topic so far, the prevalence of peripheral arterial involvement cannot be determined accurately, but based on the available literature data it tends to be relatively rare.

Gaertner et al. (15) screened 15 patients with *FBN1* mutations and they detected peripheral aneurysms in 10 of these patients, but none of them appeared in the internal mammary arteries. Bilateral popliteal aneurysms in a patient with a novel *FBN1* mutation were reported by Mohammad et al. (16), and Ghonem et al. (17) described a left subclavian artery aneurysm in a genetically confirmed MFS patient.

There are also case reports covering peripheral arterial involvement in which the diagnosis of MFS is based on clinical features or on genetic testing to exclude some of the genes responsible for related disorders.

As related syndromes of MFS often show similar clinical features, differential diagnosis is often difficult without genetic testing (12). For example Loeys-Dietz syndrome (LDS) is a MFS-related disorder, and it carries an increased risk of developing aneurysms of peripheral arteries (18).

Yetman et al. found that around one-third of adult patients with MFS had distal/peripheral aneurysms. They did not carry out genetic testing for the *FBN1* gene, they only screened the *TGFBRI* and *TGFBRII* genes to exclude the presence of LDS (19). However, additional genes like *TGFB2, TGFB3, SMAD2*, and *SMAD3* are also associated with LDS (18), thus in this case the diagnosis of LDS cannot be excluded. The importance of genetic screening can be also highlighted by another case. A case report on peripheral aneurysm formation has been presented where the patient had the diagnosis of MFS prior to genetic testing, however gene sequencing altered the diagnosis to LDS (20).

In summary, larger studies will be required in the future to make a reliable estimation of the frequency of peripheral aneurysms in MFS, for which case reports, like the present one, can provide a good starting point.

To our knowledge, just around 30 cases of true IMA aneurysms have been reported in the literature and they presented with various etiologies like MFS, LDS, Kawasaki disease, polyarteritis nodosa, atherosclerosis, systemic lupus erythematosus, etc., and few of them were idiopathic (Table 1). Only six of these were described in patients with MFS (Table 1), of which only one case report presented a MFS patient with a detected variant in the *FBN1* gene (40).

**Table 1:** Published true aneurysms of internal mammary arteries.

**References**	**Affected artery**	**Etiology**	**Treatment**
Otter et al. (21)	LIMA	Unknown	Open ligation
Giles et al. (22)	Bilateral IMA	Polyarteritis nodosa	Bilateral resection
Ishiwata et al. (23)	Bilateral IMA	Kawasaki arteritis	Open ligation
Wildhirt et al. (24)	RIMA	Atherosclerosis	Open ligation and resection
Connery et al. (25)	RIMA	Arterial fibromuscular dysplasia	Division of IMA
Tabata et al. (26)	RIMA	Atherosclerosis	Root ligation
Chan et al. (27)	LIMA	Idiopathic	Embolization
Phan et al. (28)	LIMA	EDS	Ligation
Common et al. (29)	LIMA	**MFS (clinical diagnosis)**	Coil embolization
Kugai et al. (30)	LIMA	Atherosclerosis	Aneurysmectomy and reconstruction
Engelke et al. (31)	LIMA	SLE	Coil embolization
Urso et al. (32)	RIMA	Neurofibromatosis 1	Ligation
Rose et al. (10)	LIMA	**MFS (clinical diagnosis)**	Coil embolization
Okura et al. (33)	RIMA	Idiopathic	Open ligation
Ohman et al. (12)	RIMA	LDS	Coil embolization
Awais et al. (11)	Bilateral IMA	**MFS (clinical diagnosis)**	Failed embolization, surveillance
Lindblom et al. (34)	LIMA	Idiopathic	Endovascular coiling
Heyn et al. (35)	LIMA	Idiopathic	Open resection
Alhawasli et al. (8)	Bilateral IMA	**MFS (clinical diagnosis)**	Covered stent exclusion
Chandra et al. (36)	LIMA	SLE	Coil embolization
Burke et al. (37)	LIMA	LDS, *SMAD3* mutation	Coil embolization
Piffaretti et al. (38)	LIMA	Sneddon syndrome	Stent graft
Almerey et al. (39)	RIMA	Idiopathic	Coil embolization
Nevidomskyte et al. (9)	RIMA	LDS, *SMAD3* mutation	Coil embolization
Fujiyoshi et al. (40)	Bilateral IMA	**MFS** ***FBN1*** **mutation**	Coil embolization
Mertens et al. (41)	Bilateral IMA	**MFS (clinical diagnosis)**	Coil embolization
Chen et al. (42)	Bilateral IMA	COL5A1 mutation, fibromuscular dysplasia	Coil embolization
Miyazaki et al. (43)	RIMA	Idiopathic (after aortic dissection)	Thoracoscopic resection

*So far internal mammary artery aneurysm was described in only one Marfan syndrome patient with confirmed FBN1 mutation. The bold values demonstrate the true aneurysms of the internal mammary arteries reported in Marfan syndrome patients*.

Some of the IMA aneurysms reported in MFS patients had remarkable sizes. The patient in the case report by Alhawasli et al. (8) had a 3.5 × 3.4 cm aneurysm on the proximal- and a 2 × 2 cm aneurysm on the middle part of the RIMA, along with a bilobed aneurysm measuring 1.5 × 1.5 cm proximally and 1.3 × 1.2 cm distally on the LIMA. A LIMA aneurysm measuring 6 × 4.6 cm presented with acute symptoms in the report by Rose et al. (10). Mertens et al. (41) demonstrated the occurrence of a 2.8 cm RIMA- and a 2.2 cm LIMA aneurysm in a patient.

The above case reports presented only limited information on the morphology and structure of the mammary artery aneurysm. Therefore, we conducted tortuosity measurements on IMAs of our patient. Arterial tortuosity describes the morphology of an artery with increased number or increased amplitude of curvatures. It is a common feature in LDS and arterial tortuosity syndrome, and it is also a manifestation of MFS (44). Moreover, increased vessel tortuosity has been associated with more severe aortic involvement in patients with MFS (14, 45, 46), demonstrating its potential prognostic value. Certain metrics have been described to quantify arterial tortuosity. Distance metric (DM) is the ratio of the actual path length to the linear distance of the endpoints, with the disadvantage of being insensitive to the frequency of the curves. It can be overcome by multiplying the DM with the inflection points giving rise to the inflection count metric (ICM). DM and ICM have been shown to be effective in detecting type I tortuosity abnormalities, which is characterized by high amplitude, low frequency curves. However, they are not capable of handling tight coils, for which sum of angles metric (SOAM) has been developed as a solution. SOAM tends to be increased in the presence of high frequency curves (47). The relative contribution of amplitude and frequency to the tortuosity can be assessed by a fourth metric, ICM/SOAM (14).

In the presented case, DM was increasing in the LIMA and RIMA throughout the follow-up period, indicating an increasing tortuosity. In case of the thoracic aorta, a rise of the ICM, SOAM and ICM/SOAM metrics could have been observed, demonstrating a progression in the tortuosity of the aorta, with high amplitude and high frequency curves.

The exact cause of the development of IMA aneurysms in our patient cannot be identified with certainty. He underwent cardiac surgery before the presentation of IMA aneurysms, however, the causative role of the surgery is highly questionable as the aneurysms only started to develop 6 years after the procedure at the age of 36 years, which could be the natural course of the disease. Even if nearly all the IMA aneurysms in MFS reported in the literature appeared years after cardiac surgery (8, 10, 11, 40), the connection between cardiac surgery and aneurysm formation is yet undetermined. In the reported case of Mertens et al. (41), the aneurysms presented at the age of 39 years without prior surgical or endovascular intervention.

As IMA aneurysms are extremely rare, there are no accepted guidelines on their treatment. Despite the majority of the six cases reported successful treatment with endovascular technique, there is not enough experience with managing these aneurysms in MFS. Most case reports demonstrate prompt intervention after detecting the aneurysms. However, one article reported on a failed intervention attempt in a MFS patient, where a right middle cerebral artery infarct occurred most likely secondary to an iatrogenic thromboembolic event. After this, the aneurysm was only followed-up without carrying out any further intervention. In this case MFS was not confirmed with genetic testing (11).

In the only reported case with genetically confirmed MFS, Fujiyoshi et al. (40) applied a coil embolization technique right after identifying the aneurysms, while we opted for monitoring our patient with blood pressure control due to his asymptomatic state, tortuous IMAs and prior cardiac surgery, with the aim of avoiding the possible complications of an intervention.

We believe that CT surveillance and blood pressure management in case of IMA aneurysms in MFS patients can be a suitable option in selected cases. This approach is supported by our result that the IMA aneurysms did not show any progression 32 months after their discovery.

As MFS patients are recommended to undergo timely CT imaging, it may be advisable to pay attention to the presence of peripheral aneurysms, including that of the internal mammary arteries during surveillance CT imaging.

We acknowledge, that the limitation of the current case report is the relatively short follow-up time; however, no progression of the aneurysms could be observed 32 months after their initial discovery.

## Conclusion

To our knowledge, this is the first report of IMA aneurysms with this special pearl-string-like morphology and with the analysis of tortuosity of the affected arteries and the thoracic aorta in genetically confirmed MFS patients. Furthermore, as we are concerned, this is the first case report of IMA aneurysms where follow-up was chosen as the primary approach. We also emphasize the need for genetic testing to enable us to estimate the true prevalence of IMA aneurysms in MFS patients. To do this, we recommend the use of multi-gene panel testing to establish the correct diagnosis, which enables the initiation of appropriate patient management. We believe that follow-up of IMA aneurysms with CT scan and blood pressure management can be a feasible approach in patients with MFS in selected cases.

## Data Availability Statement

The raw data supporting the conclusions of this article will be made available the corresponding author on reasonable request.

## Ethics Statement

Our study was performed in compliance with the Declaration of Helsinki, and was approved by the Semmelweis University Regional and Institutional Committee of Science and Research Ethics (SE RKEB 72/2018). The patient provided his written informed consent to participate in this study. Written informed consent was obtained from the individual for the publication of any potentially identifiable images or data included in this article.

## Author Contributions

RS evaluated the patient's clinical data, reviewed the literature, kept in touch with the patient, and drafted the manuscript. BÁ carried out the tortuosity measurements, evaluated their results, and drafted the manuscript. BS provided the CT images and drafted the manuscript. KB, ND, BR, and BV contributed to manuscript writing and took part in the patient's management. BM revised the manuscript and helped with professional advices. MP and ZS managed the patient, carried out the cardiac procedures, followed-up the patient, revised the manuscript, and helped with professional advices. All authors agree to be accountable for the content of the work.

## Conflict of Interest

The authors declare that the research was conducted in the absence of any commercial or financial relationships that could be construed as a potential conflict of interest.

## Abbreviations

CT: computed tomography
DM: distance metric
ICM: inflection count metric
IMA: internal mammary artery
LDS: Loeys-Dietz syndrome
LIMA: left internal mammary artery
MFS: Marfan syndrome
RIMA: right internal mammary artery
SOAM: sum of angles metric.
